# Outcomes in the immediate subsequent gestation following a pregnancy complicated by previable preterm prelabor rupture of membranes

**DOI:** 10.1002/pmf2.70008

**Published:** 2025-04-10

**Authors:** Jessie Y. Li‐Barton, Jennifer Okunbor, Ronan Sugrue, Sarahn Wheeler, Matthew Grace, Sarah K. Dotters‐Katz

**Affiliations:** ^1^ Department of Obstetrics and Gynecology Duke University Durham North Carolina USA; ^2^ Department of Obstetrics and Gynecology Brown University Providence Rhode Island USA; ^3^ The Coombe University Hospital University College Dublin Dublin Ireland; ^4^ Division of Maternal Fetal Medicine, Department of Obstetrics and Gynecology Duke University Durham North Carolina USA; ^5^ Division of Maternal Fetal Medicine, Department of Obstetrics and Gynecology Vanderbilt University Nashville Tennessee USA

**Keywords:** periviable PPROM, PPROM, previable PPROM, recurrence, recurrent PPROM, recurrent preterm birth, subsequent pregnancy

## Abstract

**Objective:**

Little is known about outcomes of subsequent gestation following a pregnancy complicated by previable/periviable preterm prelabor rupture of membrane (pPPROM). The objective of this study is to describe outcomes in subsequent gestation after a pregnancy complicated by pPPROM.

**Study design:**

We performed a retrospective cohort study of the subsequent pregnancy for patients with a history of pPPROM (rupture of membranes < 23 weeks) in the previous gestation of our institution from 2013 to 2022. All pregnancies were dated by ultrasound prior to pPPROM in the initial cohort. No anomalous fetuses were seen in this cohort. Subsequent pregnancies continuing beyond 13 weeks and 6 days were included in the analysis. Our primary outcome was gestational age (GA) at delivery in the subsequent pregnancy. Secondary outcomes included recurrent preterm birth (PTB), recurrent PPROM, and recurrent pPPROM. Secondary analysis was performed comparing patients delivering preterm versus term in subsequent pregnancy, and excluding patients missing delivery data or with loss < 14 weeks.

**Results:**

Of 100 patients with pPPROM, 42 patients (42%) had a subsequent pregnancy. Out of 42, 36 (85.7%) reached second trimester, 5 (11.9%) had first trimester loss, including one ectopic pregnancy of four spontaneous abortions at < 12 weeks, and one was lost to follow‐up. Median GA at delivery in the subsequent pregnancy (*n* = 36) was 37.4 weeks (IQR 35.6, 38.9 weeks). Out of 36 patients, 10 patients (27.8%) had recurrent PTB, of whom 4 (11.1%) had recurrent PPROM and 2 (5.6%) had recurrent previable PPROM. One patient had periviable preterm labor. The median GA delivery of patients delivering preterm (*n* = 10) was 30.4 weeks (IQR 21.4, 34.3 weeks). Demographics, GA at pPPROM in the first pregnancy, and use of 17‐P and/or cerclage did not differ between those who did and did not deliver preterm in the subsequent pregnancy.

**Conclusion:**

In patients with prior pPPROM with subsequent pregnancy continuing beyond the first trimester, more than two‐thirds delivered at term in the subsequent pregnancy. However, recurrent previable PPROM occurred in 5.6% of patients. No clear risk factors for recurrent PTB (beyond PTB history) were identified in this cohort.

## 1 INTRODUCTION

Preterm prelabor rupture of membranes (PPROM), defined as membrane rupture before the onset of labor occurring prior to 37 weeks of gestation [1], occurs in 3%–4.5% of all pregnancies [2]. Previable/periviable PPROM (pPPROM), which we define here as rupture of membranes prior to 23 weeks of gestation, occurs in < 1% of all pregnancies and is associated with significant neonatal and maternal morbidity and mortality [3, 4].

In the literature, the recurrence risk of PPROM in subsequent pregnancies has revealed rates ranging from 10% to 32% [5, 6, 7, 8], with potential risk factors for recurrence being short interval pregnancy, increasing maternal age, and gestational age of PPROM in prior pregnancy. Van der Heyden et al. specifically studied the subsequent pregnancy after PPROM occurring prior to 27 weeks of gestation and demonstrated a recurrence rate of PPROM prior to 27 weeks of 9% and prior to 34 weeks of 15% [8].

There are much fewer data in the literature regarding outcomes in subsequent pregnancies following a pregnancy affected by pPPROM, which limits providers’ ability to counsel patients regarding future pregnancies in this setting. One retrospective cohort study by Monson et al. did study subsequent pregnancy outcomes after pPPROM and demonstrated a high risk of premature delivery in the subsequent pregnancy, with 46% before 37 weeks, 30% before 34 weeks, 23% before 28 weeks, and 17% before 24 weeks [9]. Of note, this study included a relatively racially homogenous cohort with almost 90% of the cohort identifying with the white race and also defined previable PPROM as < 24 weeks of gestation. In this study, we aim to describe a single institution's experience with outcomes in the subsequent gestation following one pregnancy affected by pPPROM.

## 2 MATERIALS AND METHODS

This study was approved by the Institutional Review Board (Pro00110082, 2/14/22). A retrospective review was conducted of the immediate subsequent singleton pregnancy of patients with a history of pPPROM in singleton pregnancies, defined here as rupture of membranes prior to 23 weeks, 0 days gestation in the prior gestation, who were cared for at our institution from 2013 to 2022. We define previable as prior to 20 weeks and 0 days. All patients had pregnancy dating by ultrasound prior to pPPROM and presented not in active labor. Included patients must have reached > 14 weeks, 0 days of gestation in the subsequent pregnancy and must have had delivery data available in our system. Patients with pregnancies that did not extend beyond 13 weeks and 6 days or missing delivery data were excluded. This cohort did not include any pregnancies with fetal anomalies in either the initial or subsequent pregnancy. Management of the subsequent pregnancy occurred with maternal fetal medicine at a single academic tertiary center. Use of 17‐P and/or cerclage was based on guidelines from the American College of Obstetrics and Gynecology and the Society for Maternal Fetal Medicine at the time of the subsequent pregnancy.

The primary outcome measured in this study was gestational age at delivery in the immediate subsequent pregnancy. Secondary outcomes included recurrent preterm delivery < 37 weeks of gestation, recurrent PPROM, and recurrent pPPROM.

Secondary analysis was performed comparing patients with recurrent preterm birth to those delivering at term in order to attempt to identify factors associated with recurrent preterm birth after pPPROM. This analysis also excluded patients lost to follow up and those with first trimester loss. Descriptive statistics were used to describe patient characteristics and were compared between patients who delivered at term and who delivered preterm in the immediate subsequent pregnancy using *t*‐tests for continuous variables and Fisher's exact test for categorical variables. All statistical analyses were performed using STATA version 18.

## 3 RESULTS

Of 100 patients identified within our system with pPPROM during the study period (from 2013 to 2022), 42 patients had a subsequent pregnancy. Of these patients, five patients had first trimester losses, with four patients having spontaneous abortions, and one patient with an ectopic pregnancy, and one was lost to follow up. A total of 36 patients reached the second trimester in the subsequent pregnancy and had delivery data available for review.

**FIGURE 1:**
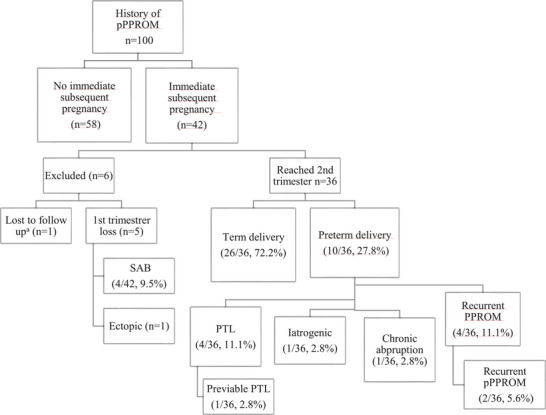
Flow chart of pregnancy outcomes for patients with a history of previable/periviable PPROM.

The median gestational age at delivery in the subsequent pregnancy was 37.4 weeks (IQR: 35.6, 38.9 weeks). Figure 1 demonstrates outcomes in the immediate subsequent pregnancy after pPPROM. A total of 10 patients (27.8%) had a preterm delivery in the subsequent pregnancy. The median gestational age at delivery among these patients with preterm delivery was 30.4 weeks (IQR: 21.4, 24.3 weeks). One patient was iatrogenically delivered preterm at 36 weeks in the setting of prior classical cesarean. Four patients (11.1%) had recurrent PPROM. Of these, two patients (5.6%) had recurrent previable PPROM (18 and 17 weeks); the other two delivered at 22/5 and 29/5 weeks. Four patients (11.1%) had spontaneous preterm labor (one at 21 weeks), and one patient delivered preterm in the setting of chronic abruption.

The only statistically significant difference in demographics and characteristics between patients who had a preterm delivery and those who had a term delivery was a history of preterm birth prior to the pPPROM pregnancy (*p* = −0.01) (Table 1). Gestational age of pPPROM was not associated with recurrent preterm delivery (*p* = 0.43). Furthermore, neither 17‐OHP (p = 0.20) nor cerclage placement (p = 0.45) were associated with differences in recurrent preterm delivery (Table 1).

**TABLE 1:** Demographics and pregnancy management for patients delivering at term compared to those delivering preterm in the subsequent pregnancy after pregnancy complicated by previable/periviable PPROM.

	All included patients (*N* = 36)	Delivered at term *N* = 26 (%)	Delivered preterm *N* = 10 (%)	*p*
Median maternal age, yrs (IQR)	30.0 [21.0, 33.0]	29.5 [21.0, 33.0]	31.0 [24.0, 35.0]	0.49
Race^a^				0.22
White	3 (8.3)	3 (11.5)	0 (0.0)	
Black	22 (61.1)	17 (65.4)	5 (50.0)	
Other	4 (11.1)	3 (11.5)	1 (10.0)	
Hispanic/Latinx	7 (19.4)	3 (11.5)	4 (40.0)	
Insurance				0.82
Self‐pay	1 (2.8)	1 (3.8)	0 (0.0)	
Medicaid	18 (50.0)	13 (50.0)	5 (50.0)	
Private	16 (44.4)	12 (46.2)	4 (40.0.)	
Missing	1 (2.8)	0 (0.0)	1 (10.0)	
cHTN	0 (0.0)	0 (0.0)	0 (0.0)	>0.99
History of PTB (prior to incident PPROM)	7 (19.4)	2 (7.7)	5 (50.0)	0.01
Diabetes	4 (11.4)	4 (15.4)	0 (0.0)	0.21
Asthma	4 (11.4)	3 (11.5)	1 (10.0)	0.90
Depression	5 (13.9)	3 (11.5)	2 (20.0)	0.51
Tobacco use	4 (11.4)	4 (15.4)	0 (0.0)	0.19
GA of PPROM in initial pregnancy, weeks (IQR)	20.4 [19.1, 22.2]	20.4 [19.6, 22.1]	20.4 [17.3, 22.3]	0.43
Median BMI at admission (IQR)	31.2 [25.7, 35.8]	32.5 [27.0, 36.8]	29.9 [21.0, 32.9]	0.23
Recurrent PPROM	4 (0.11)	0 (0.0)	4 (40.0)	0.004
Received 17‐P in subsequent pregnancy	9 (25.0)	5 (19.2)	4 (40.0)	0.20
Received cerclage in subsequent pregnancy	11 (30.6)	7 (26.9)	4 (40.0)	0.45

^a^ We recognize that race has no genetic basis, and is better considered as a proxy for racism.

## 4 DISCUSSION

pPPROM is a rare but devastating pregnancy complication that carries substantial risks for neonatal and maternal morbidity and mortality. Because patients with pPPROM have been excluded from many preterm birth trials, clear guidelines regarding management of this population remain ambiguous. In particular, counseling this patient population about their future pregnancies is challenging with very limited data to guide this discussion.

This study sought to elucidate outcomes in an immediate subsequent pregnancy following a gestation affected by pPPROM. In our cohort, about a third of patients experienced recurrent preterm delivery in their subsequent pregnancy, with 12% having recurrent PPROM and 9% experiencing recurrent pPPROM. This rate of recurrent preterm delivery is comparable to the risk of recurrent preterm birth among patients following singleton pregnancies with spontaneous preterm birth < 37 weeks, estimated to be about 30% in a systematic review with meta‐analysis [10], though this population was noted to have only 7% of patients with PPROM. It is notable and not surprising that those who had additional preterm births aside from the pPPROM pregnancy had higher rates of subsequent preterm birth. Monson et al. also described a retrospective cohort of patients with a history of pPPROM and found a slightly higher rate of recurrent preterm delivery in the immediate subsequent pregnancy of 46% [9]. We did not identify any clear risk factors (beyond a history of preterm birth) for recurrent preterm delivery in this cohort.

We believe that our cohort is an important addition to the literature given a highly diverse population that is cared for within our large referral area. Racial differences in preterm birth rates are well‐described in the literature, with disparities persisting despite accounting for known preterm birth risk factors, such as socioeconomic status, education level, and smoking [11, 12], suggesting non‐sociodemographic etiologies for these disparities. These differences are also reflected when specifically observing the previable/periviable time period [13].

Different methods to prevent recurrent preterm deliveries are reflected in our cohort. Intramuscular 17‐OHPC was previously recommended for preterm birth prophylaxis before being withdrawn from the market by the FDA in 2023 given no data to support efficacy for prevention of recurrent preterm birth. In our cohort, 27% of patients received 17‐OHPC. Additionally, 30% of patients underwent cerclage placement in their subsequent pregnancy. While history‐indicated cerclage is classically recommended for patients with features of cervical insufficiency, it has been considered in some settings for patients with a history of pPPROM—however, our study did not show any difference in recurrent preterm birth with cerclage placement. This is in line with findings of other retrospective cohorts in the literature [9, 14]. Bart et al., who studied a retrospective cohort of 74 patients with a history of pPPROM, actually demonstrated higher rates of recurrent preterm birth for those who underwent cerclage placement compared to those who did not [14]. Certainly, these studies are limited by small sample sizes and heterogeneity in patient population and larger‐scale studies are needed to draw definitive conclusions. However, based on this limited data, a recent SMFM Consult Series about management of pPPROM recommends reserving history‐indicated cerclage only for those with classic features of cervical insufficiency or unexplained second trimester loss without evidence of placental abruption [15].

This study is certainly limited as a retrospective experience of a single center with a small sample size, reflecting the overall rarity of pPPROM. We also did not assess the interpregnancy interval, which may impact the rate of recurrent preterm birth. While all patients at our center were counseled about options for management in subsequent pregnancy, guidelines for progesterone usage changed during the study period. At our center, for patients without a clear history of cervical insufficiency but early PPROM, we offer cerclage or serial cervical length screening. Thus, outcomes may have been somewhat impacted by patient choice. Additionally, the small numbers in our study increase the risk of Type 2 error. Finally, patients may have had a subsequent pregnancy outside our health system, the outcomes of which we do not have access to/would not be included in this study. Though, as a large referral center, if there was an extremely aberrant outcome, it is most likely that the patient would have been transferred for NICU proximity. However, we believe a strength of our cohort is its representation of a highly diverse population drawn from a large referral area in North Carolina. Furthermore, we believe that our findings contribute to the very limited data available regarding this specific, high‐risk population, for which it is very unlikely that sufficiently powered trials are possible.

## 5 CONCLUSION

In summary, patients affected by pPPROM are at elevated risk of recurrent preterm delivery in the immediate subsequent pregnancy, though our findings demonstrate this risk may be similar to patients who have previously experienced spontaneous preterm birth at less than 37 weeks. Aside from a history of preterm birth, we did not identify any risk factors associated with recurrent preterm delivery. There is a paucity of data in the literature to guide management in this specific patient population, and we hope that our findings may be helpful in counseling patients regarding their risks in subsequent pregnancy.

## CONFLICT OF INTEREST STATEMENT

The authors declare no conflicts of interest.

## References

[1] Siegler Y. , Z. Weiner , and I. Solt . 2020. “ACOG Practice Bulletin No. 217: Prelabor Rupture of Membranes.” Obstetrics and Gynecology 136(5): 1061. 33093409 10.1097/AOG.0000000000004142

[2] Lee T. , and H. Silver . 2001. “Etiology and Epidemiology of Preterm Premature Rupture of the Membranes.” Clinics in Perinatology 28(4): 721–34. 11817185 10.1016/s0095-5108(03)00073-3

[3] Can E. , S. C. Oglak , and F. Olmez . 2022. “Maternal and Neonatal Outcomes of Expectantly Managed Pregnancies With Previable Preterm Premature Rupture of Membranes.” Journal of Obstetrics and Gynaecology Research 48(7): 1740–9. 35411577 10.1111/jog.15239

[4] Simons N. E. , A. A. de Ruigh , L. I. van der Windt , B. M. Kazemier , A. G. van Wassenaer‐Leemhuis , A. S. van Teeffelen , E. van Leeuwen , B. W. Mol , J. van 't Hooft , and E. Pajkrt . 2021. “Maternal, Perinatal and Childhood Outcomes of the PPROMEXIL‐III Cohort: Pregnancies Complicated by Previable Prelabor Rupture of Membranes.” European Journal of Obstetrics, Gynecology, and Reproductive Biology 265: 44–53. 34428686 10.1016/j.ejogrb.2021.08.007

[5] Getahun D. , D. Strickland , C. V. Ananth , M. J. Fassett , D. A. Sacks , R. S. Kirby , and S. J. Jacobsen . 2010. “Recurrence of Preterm Premature Rupture of Membranes in Relation to Interval Between Pregnancies.” American Journal of Obstetrics and Gynecology 202(6): 570 e1–6. 10.1016/j.ajog.2009.12.010 20132922

[6] Asrat T. , D. F. Lewis , T. J. Garite , C. A. Major , M. P. Nageotte , C. V. Towers , D. M. Montgomery , and W. A. Dorchester . 1991. “Rate of Recurrence of Preterm Premature Rupture of Membranes in Consecutive Pregnancies.” American Journal of Obstetrics and Gynecology 165(4 Pt 1): 1111–5. 1951524 10.1016/0002-9378(91)90481-6

[7] Lee T. , M. W. Carpenter , W. W. Heber , and H. M. Silver . 2003. “Preterm Premature Rupture of Membranes: Risks of Recurrent Complications in the Next Pregnancy Among a Population‐Based Sample of Gravid Women.” American Journal of Obstetrics and Gynecology 188(1): 209–13. 12548219 10.1067/mob.2003.115

[8] van der Heyden J. L. , S. M. van Kuijk , D. P. van der Ham , K. J. Notten , T. Janssen , J. G. Nijhuis , C. Willekes , et al. 2013. “Subsequent Pregnancy After Preterm Prelabor Rupture of Membranes Before 27 Weeks' Gestation.” American Journal of Perinatology Reports 3(2): 113–8. 24147248 10.1055/s-0033-1353389 PMC3799705

[9] Monson M. A. , K. J. Gibbons , M. S. Esplin , M. W. Varner , and T. A. Manuck . 2016. “Pregnancy Outcomes in Women With a History of Previable, Preterm Prelabor Rupture of Membranes.” Obstetrics and Gynecology 128(5): 976–82. 27741176 10.1097/AOG.0000000000001682 PMC5774863

[10] Phillips C. , Z. Velji , C. Hanly , and A. Metcalfe . 2017. “Risk of Recurrent Spontaneous Preterm Birth: A Systematic Review and Meta‐Analysis.” BMJ Open 7(6): e015402. 10.1136/bmjopen-2016-015402 PMC5734267 28679674

[11] Manuck T. A. . 2017. “Racial and Ethnic Differences in Preterm Birth: A Complex, Multifactorial Problem.” Seminars in Perinatology 41(8): 511–8. 28941962 10.1053/j.semperi.2017.08.010 PMC6381592

[12] Johnson J. D. , C. A. Green , C. J. Vladutiu , and T. A. Manuck . 2020. “Racial Disparities in Prematurity Persist among Women of High Socioeconomic Status.” American Journal of Obstetrics & Gynecology Maternal‐Fetal Medicine 2(3): 100104. 10.1016/j.ajogmf.2020.100104 PMC7654959 33179010

[13] DeFranco E. A. , E. S. Hall , and L. J. Muglia . 2016. “Racial Disparity in Previable Birth.” American Journal of Obstetrics and Gynecology 214(3): 394 e1–7. 10.1016/j.ajog.2015.12.034 26721776

[14] Bart Y. , M. Fishel Bartal , R. Plaschkes , D. Sebag , S. P. Chauhan , B. M. Sibai , R. Meyer , E. Kassif , R. Yoeli , and S. Mazaki‐Tovi . 2024. “The Role of Cerclage in Subsequent Pregnancy Following Previable Prelabor Rupture of Membranes.” American Journal of Perinatology 41(S 01): e1397–1403. 36746399 10.1055/a-2028-7633

[15] Society for Maternal‐Fetal M , Battarbee A. N. , S. S. Osmundson , A. M. McCarthy , J. M. Louis , and SMFM Publications Committee . 2024. “Society for Maternal‐Fetal Medicine Consult Series #71: Management of Previable and Periviable Preterm Prelabor Rupture of Membranes.” American Journal of Obstetrics and Gynecology 231(4): B2–15. 10.1016/j.ajog.2024.07.016 39025459

